# An Accurate, Rapid and Cost-Effective Method for T-nos Detection Based on CRISPR/Cas12a

**DOI:** 10.3390/foods12030615

**Published:** 2023-02-01

**Authors:** Yuling Wang, Cheng Peng, Lin Ding, Zhixun Su, Xiaoyun Chen, Xiaofu Wang, Meihao Sun, Junfeng Xu

**Affiliations:** 1 College of Life Sciences, Zhejiang Normal University, Jinhua 321004, China; 2 State Key Laboratory for Managing Biotic and Chemical Threats to the Quality and Safety of Agro-Products, Zhejiang Academy of Agricultural Sciences, Hangzhou 310021, China; 3 College of Food and Pharmaceutical Sciences, Ningbo University, Ningbo 315800, China

**Keywords:** T-nos, nucleic acid detection, CRISPR/Cas12a, fluorescence visualisation, PCR

## Abstract

CRISPR/Cas12a technology is used for nucleic acid detection due to its specific recognition function and non-specific single-stranded DNA cleavage activity. Here, we developed a fluorescence visualisation detection method based on PCR and CRISPR/Cas12a approaches. The method was used to detect the nopaline synthase terminator (T-nos) of genetically modified (GM) crops, circumventing the need for expensive instruments and technicians. For enhanced sensitivity and stability of PCR-CRISPR/Cas12a detection, we separately optimised the reaction systems for PCR amplification and CRISPR/Cas12a detection. Eleven samples of soybean samples were assessed to determine the applicability of the PCR-CRISPR/Cas12a method. The method could specifically detect target gene levels as low as 60 copies in the reaction within 50 min. In addition, accurate detection of all 11 samples confirmed the applicability. The method is not limited by large-scale instruments, making it suitable for mass detection of transgenic components in plants in the field. In conclusion, we developed a new, accurate, rapid, and cost-effective method for GM detection.

## 1. Introduction

Transgenic technology has arisen in response to the pressing demand for more practical and effective agricultural production techniques to increase yields to meet the rapid growth of the human population, and the resulting changing climate. New plant types created using transgenic technology possess notable advantages in terms of yield, quality, disease resistance, and production cost. The International Service for the Application of Agrobiotechnology’s 2019 Annual Report (ISAAA, 2019) states that genetically modified (GM) crops have been used in 70 countries worldwide, with a total cultivated area of 190.4 million hectares. More than 40 countries worldwide have enacted systems for the labelling of GM plant cultivation and associated agricultural products [1,2,3], requiring the detection of GM components in plants and their processed products [4]. As foreign genetically modified crops are imported as commodities, the public’s awareness of genetically modified crops has gradually improved. China adopts a compulsory labelling system for goods containing genetically modified ingredients or made from genetically modified organisms. Therefore, GM detection technology is essential for the development of rapid, effective, affordable GM detection methods to protect China’s agricultural import and export trade, meet biosafety regulatory needs, and support GM labelling management systems.

Current transgenic assays rely on in-laboratory amplification [5], which includes conventional PCR, quantitative PCR (qPCR), and thermostatic amplification techniques such as recombinase polymerase amplification (RPA) and loop-mediated isothermal amplification (LAMP). Conventional PCR has been the main method of nucleic acid amplification since its emergence. It is inexpensive, has a simple primer design, a mature amplification system has been established in the laboratory, and it is used as one of the main methods for transgenic detection. However, its amplification duration is long, and results cannot be directly observed, instead relying on agarose gel electrophoresis and fluorescent staining [6]. Real-time PCR is another important transgenic assay that is specific, and results can be observed and quantified in real time [1], but it is limited by the need for large instruments, the high cost of detection, and the requirement for dedicated laboratory facilities.

RPA and LAMP are emerging technologies [7,8,9,10,11,12] that can amplify thermostatically, have short amplification times, and are not limited by reaction instrumentation. However, kits for thermostatic amplification are expensive, primer design is complex, extensive screening is required [13], and they are not suited to high-volume transgenic assays. In order to overcome this, and allow rapid transgenic detection and visual observation, we are combining conventional PCR products with a clustered regularly interspaced short palindromic repeats (CRISPR) system, so that transgenic detection can be observed simply outside the laboratory on an inexpensive basis.

The clustered regularly interspaced short palindromic repeats/CRISPR-associated enzyme system (CRISPR/Cas) is an RNA-induced acquired immune system consisting of CRISPR sequences with a family of Cas genes that encode proteins that function as nucleases and can specifically recognise DNA sequences for cleavage [14]. Taking advantage of this property, a new generation of rapid molecular assays such as SHERLOCK and DETECTR have been developed [15,16,17], which are widely used for specific and sensitive detection of nucleic acids. The Cas12a enzyme, guided by its guide RNA, not only cleaves the target, but also indiscriminately cleaves single-stranded DNA [18]; hence, it is limited by sensitivity and cannot be used alone for nucleic acid detection, and cumbersome amplification-free detection steps are needed [19,20]. Therefore, nucleic acid detection can be effectively carried out by combining the CRISPR approach with amplification technology.

**Figure 1:**
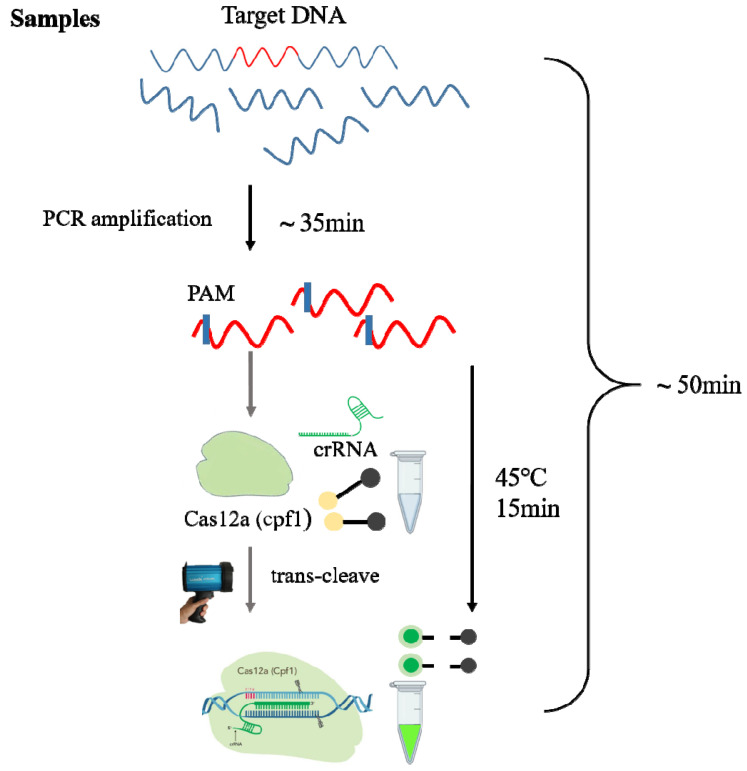
A T-nos detection method combining conventional PCR amplification with the CRISPR/Cas12a assay. The target DNA is specifically amplified by conventional PCR, and a crRNA guide sequence is specially designed targeting a region in the target DNA. The amplified product is added to the CRISPR bulk assay, which activates the trans-cleavage function of the Cas12a enzyme, resulting in cleavage of the quenched fluorescent ssDNA reporter if the target DNA is present, and hence in-tube fluorescence.

In this study, the T-nos from *Agrobacterium tumefaciens* nopaline synthase was selected, since it is often used as an indicator of the presence of transgenic components in an assay, and the P-35S and T-nos coverage in all authorised GM organisms worldwide is >70% [21]. Conventional PCR is widely used for the detection of transgenic components due to its low contamination susceptibility, low detection cost, and simple primer design [2]. In this study, we combined conventional PCR amplification with the CRISPR/Cas12a assay to detect T-nos components in plant DNA. If the target sequence is present in the amplified product, it can bind to crRNA and activate the trans-cleavage function of the cas12a enzyme, which cleaves the probe, generating fluorescence. The fluorescence can be observed visually using a portable fluorescent excitation lamp to determine the presence or absence of transgenic components. Highly efficient amplification primers were selected to reduce the reaction time and increase sensitivity via system optimisation and reducing the number of PCR cycles. After determining the sensitivity and specificity, the established assay was tested using transgenic samples and the results were compared with qPCR to systematically assess the applicability of the method. The assay could achieve inexpensive, simple, rapid, non-contaminating, and intuitive transgene detection within 1 h (Figure 1).

## 2. Materials and Methods

### 2.1. Materials

LbCas12a (Lachnospiraceae bacterium ND2006) was purchased from Bio-Lifesci (Guangzhou, China). Positions1847–2099 of the T-nos sequences (GenBank no. V00087) were selected as the target region for primer design and CRISPR detection. The primers and three crRNAs were designed using DNAMAN software (Version 6.0, Lynnon Biosoft, San Ramon, CA, USA) (Table S1). Three crRNAs were synthesised by Bio-Lifesci. Primers, qPCR Taqman probes, fluorescent CRISPR reporter probes, NOS full-length plasmid DNA, and a series of NOS fragments were synthesised by Sangon Biotech (Shanghai, China) (Table S1). Conventional PCR reagents were purchased from TaKaRa, PCR primers were synthesised by Shanghai Biotech (Table S1), Agarose (low electroosmotic), 10 × TBE; Premixed Powder; 4S Gel-red Nucleic Acid Dye (Sangon Biotech, Shanghai, China). GM soybean (ZUTS-33, transgenic *g10-epsps* gene) was provided by Zhejiang University (Hangzhou, China). Non-GM rice, cotton, canola, corn, and soybean were purchased from a local market. DNA was extracted using the Plant DNA kit (QIAGEN, Dusseldorf, North Rhine-Westphalia, Germany) and quantified using Nanodrop 2000 (Thermo Fisher Scientific, Waltham, MA, USA). Since the unit measured by Nanodrop is ng/μL, we can convert it into copy per microliter using the following formula: copies/μL = (6.02 × 10^23^ copies/mol) × (ng/μL × 10^−9^)/(DNA length × MW). MW stands for average molecular weight.

### 2.2. Optimisation of Conventional PCR Methods

PCR amplification was performed using the high-fidelity enzyme KOD FX (Takara). A Biometra TAdvanced 96 SG (Biometra, Germany) instrument was used for conventional PCR. The concentration of primers in the final reaction system was 0.4 μM each, 0.5 mM dNTPs, KOD FX enzyme concentration 0.02 unit/μL, and 2 μL DNA template (2.4 × 10^3^ copies/μL, 9 × 10^4^ copies/μL T-nos plasmid DNA and 5-fold serial dilution, 1.88 × 10^4^ copies/μL GM soybean DNA and 5-fold serial dilution) was added to the 23 μL amplification system resulting in a total reaction volume of 25 μL. Unless otherwise stated, the PCR reaction procedure was: pre-denaturation at 95 °C for 2 min, then 29 cycles of 98 °C for 10 s, 59 °C for 15 s, and 68 °C for 10 s (reaction time was about 35 min), and the PCR was used for CRISPR/Cas12a reaction directly after the end of PCR. For product validation, 8 μL of PCR products were used with EPS301 Gel Electrophoresis System (South San Francisco, CA, USA) and electrophoresed on a 1.5% agarose gel and then photographed with a ZF-258 Fully Automatic Gel Imaging Analysis System (Shanghai Jiapeng, China). The annealing temperatures were set to 56 °C, 57 °C, 58 °C, 59 °C, 60 °C, 61 °C, 62 °C, and 63 °C, respectively, using 2.4 × 10^3^ copies/μL plasmid DNA as the template. After determining the optimum primers, the primer concentrations were set to 0.1 μmol/L, 0.2 μmol/L, 0.3 μmol/L, 0.4 μmol/L, and 0.5 μmol/L, and the amplification products were detected by 1.5% agarose gel electrophoresis, and the best primer concentration and annealing temperature was the one with brighter amplified bands.

### 2.3. The Cas12a Bulk Assay

Unless otherwise stated, the system contained 100 nM of Cas12a, 200 nM of crRNA, and 2 μM of fluorescent reporter probe. In a typical 20 μL system, the above components were mixed in 18 μL 1 × CRISPR reaction buffer solution, plus 2 μL of amplification product. The reaction mixture was incubated in a BioTek Cytation5 multifunctional microplate reader (BioTek, Winooski, VT, USA) for 45 min at 45 °C, while fluorescence was measured every 1 min. The fluorescence visible to the naked eye is to add the reactant to the test tube and incubate for 15 min at a constant temperature to visually observe the end point. Fluorescence was observed using the LUYOR-3415RG hand-held Light-Emitting Diode (LED) blue light illuminator (Luyor, Santa Barbara, CA, USA). Signals were identified with the naked eye and recorded on a smartphone.

### 2.4. Statistics and Reproducibility

We used Excel (2016, Microsoft) to plot the fluorescence curves of CRISPR reaction. The fluorescence signal at 5 min intervals was taken as the vertical coordinate. Each experiment was repeated three times.

### 2.5. Real-Time PCR Studies to Detect NOS

Plasmids and GM plant samples were quantitatively analysed using real-time PCR. Samples were analysed and identified according to current GMO testing standard parameters and quoted the sequence of Yang et al. (DOI: 10.1002/jsfa.2193) [22]. Our reaction system comprised 1 × Faststart Essential DNA Probes Master (Roche, Mannheim, BW, Germany), 0.4 μM forward primer, 0.4 μM reverse primer, 0.2 μM probe, and 2 μL target DNA (1.88 × 10^4^ copies/μL GM soybean DNA and 5-fold serial dilution) to make a total reaction volume of 25 μL. The reaction mixture was placed in a Bio-Rad CFX96 Touch real-time PCR system (Bio-Rad, Hercules, CA, USA) and thermally cycled as follows: 95 °C for 10 min, then 95 °C for 15 s and 60 °C for 1 min over 39 cycles. The reaction was considered negative after 36 cycles.

### 2.6. Construction and Purification of Reference Plasmid DNA

For the construction and purification of reference plasmid DNA in the susceptibility assay experiment, reference plasmids and strains were obtained by cloning conserved sequences of NOS terminators into pUC-SP (Sangon Biotech, Shanghai, China). After plasmid extraction by strain, plasmid concentration was determined and the copy number was calculated using a Nanodrop spectrophotometer (Thermo Scientific, Waltham, MA, USA). The plasmid solution was diluted in TE buffer to make a 5-fold dilution gradient used to detect sensitivity.

## 3. Results

### 3.1. Optimisation of the PCR System and Amplification Conditions

**Figure 2:**
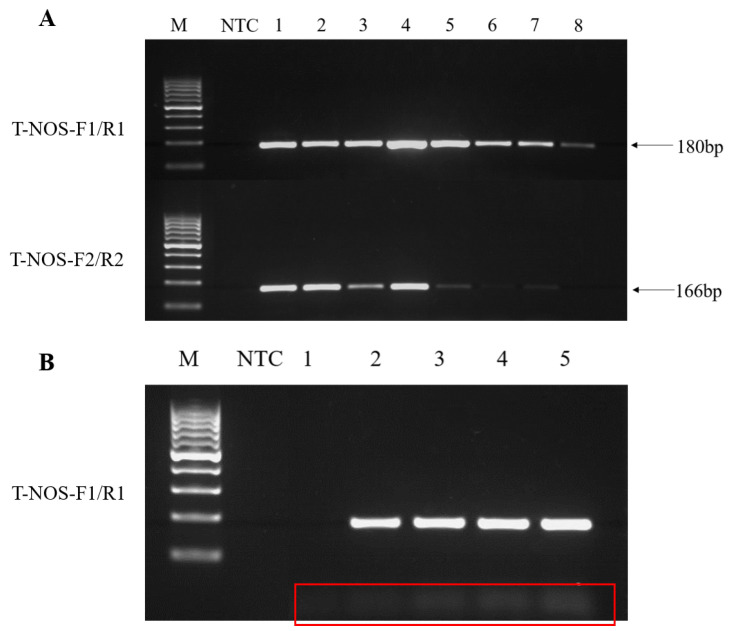
Optimisation of conventional PCR amplification assessed by agarose gel electrophoresis (AGE) conducted using 2.4 × 10^3^ copies/μL T-nos plasmid as template. (**A**) AGE visualisation using primer pairs F1/R1 and F2/R2 at different annealing temperatures. Lanes 1–8, annealing temperatures (56, 57, 58, 59, 60, 61, 62, and 63 °C). (**B**) AGE visualisation using primer pairs F1/R1 at different concentrations. Lanes 1–5 represent primer concentrations (0.1, 0.2, 0.3, 0.4, and 0.5 μmol/L). Primer dimers are indicated by a red box. M, DNA Ladder H1 (100~1000 bp); NTC, no template control.

The assay employed a plasmid DNA at 2.4 × 10^3^ copies/μL harbouring conserved T-nos sequences as a template. This concentration is the concentration of plasmid DNA selected from our pre-experiment, which is relatively suitable for primer and annealing temperature screening. The plasmid concentration was determined using Nanodrop and diluted to the target concentration with TE buffer. To screen for suitable primers, the PCR conditions for primers were optimised. Condition optimisation tests were performed with different annealing temperatures (56, 57, 58, 59, 60, 61, 62, and 63 °C). As shown in Figure 2A, the PCR amplification results showed that both primer pairs (Table S1) could amplify target bands at 2.4 × 10^3^ copies/μL template; the highest amplification efficiency was achieved at 59 °C annealing, and the band brightness of primer pair T-NOS-F1/R1 was higher than that of T-NOS-F2/R2. Based on the test results, the final primer pair selected was T-NOS-F1/R1 with a band size of 180 bp, and the chosen annealing temperature was 59 °C.

Different primer concentrations (0.1, 0.2, 0.3, 0.4, and 0.5 μM) were assessed in system optimisation tests to improve the PCR amplification system with T-NOS-F1/R1 primers. The results showed that the amplified bands became brighter with increasing primer concentration, and primer dimers were also generated (Figure 2B). After comprehensive evaluation, we chose 0.4 μM as the primer concentration. Based on the results, the optimal 25 µL reaction system comprised 1 × PCR buffer, 0.4 µM of dNTPs, 0.4 µM of upstream and downstream primer, pre-denaturation at 95 °C for 2 min, followed by 35 cycles of denaturation at 94 °C for 10 s, annealing at 59 °C for 10 s, extension at 72 °C for 5 s, a final extension at 72 °C for 5 min, and storage at 4 °C.

### 3.2. Optimisation of the In-Tube CRISPR Detection System

**Figure 3:**
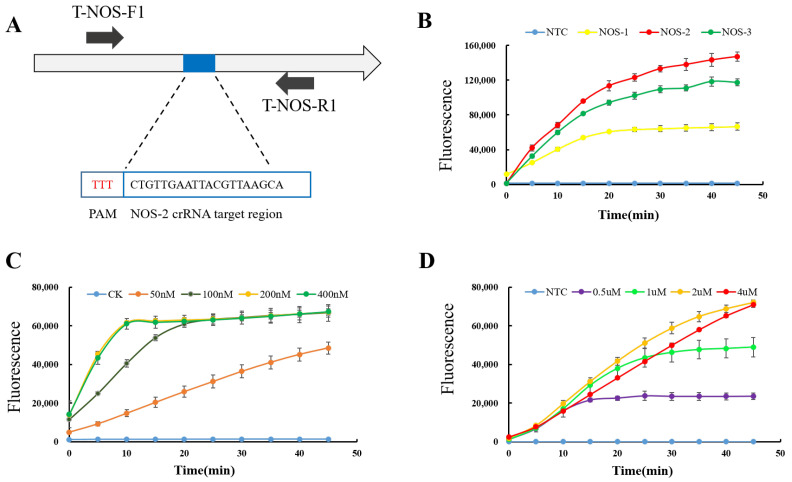
Optimisation of the CRISPR/Cas12a detection system for T-nos detection. (**A**) For crRNA design, three sites based on T-nos conserved sequences were screened, and upstream and downstream primer sites corresponding to T-NOS-F1/R1 were identified. (**B**) Cas12a assays using crRNA NOS-1, NOS-2 and NOS-3. NTC, no target DNA control. The x-axis shows the reaction time, and the y-axis shows the fluorescence signal. (**C**) Comparing the Cas12a assay using different concentrations of the same crRNA. (**D**) Comparing the Cas12a assay using different concentrations of fluorescent probes.

The detection efficiency of the CRISPR/Cas12a-based T-nos assay was improved by optimising the reaction system, including the design and screening of crRNA, the optimal concentration of crRNA, and the optimal concentration of the probe. The fluorescent Cas12a assay was performed to target T-nos plasmids in the bulk assay system by detecting fluorescence using an enzymatic standard. Initially, three T-nos target sequence sites were used to design the crRNAs (Figure 3A, Table S1). Fluorescence assays for CRISPR/Cas12a showed that crRNAs NOS-1, NOS-2, and NOS-3 interacted with the substrate target DNA, and all showed incidental single-stranded DNA cleavage. Compared with NOS-1 and NOS-3, NOS-2 displayed a stronger fluorescence signal, indicating that it may have a greater affinity for the target DNA (Figure 3B).

In addition, the effect of crRNA concentration on the detection system was tested. CRISPR reactions using in-system concentrations of 50, 100, 200, and 400 nM of the same crRNA were performed, and the results showed that 200 nM and 400 nM had similar high fluorescence signals (Figure 3C). Thus, for cost reasons, 200 nM was selected as the optimal crRNA concentration.

Finally, the effect of reporter probe concentration on the efficiency of trans-cleavage by the Cas12a enzyme was evaluated. Different concentrations of probe were cleaved and fluoresced by the activated Cas12a enzyme, with the highest fluorescence intensity observed at an in-system probe concentration of 2 μM (Figure 3D). Based on the experimental results, the final reaction system for the bulk CRISPR assay was a 20 μL system containing 1 × Reaction Buffer, 100 nM of LbCas12a, 200 nM of crRNA, 2 μM of fluorescent reporter probe, 2 μL of DNA solution, and RNase-free water.

### 3.3. Combining the CRISPR Assay with Conventional PCR, and Optimising the Reaction Time

**Figure 4:**
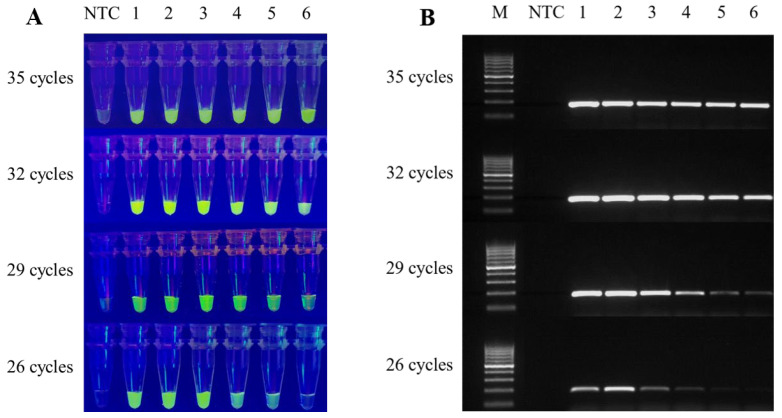
Detection of conventional PCR amplification products by the CRISPR/Cas12a system, and visualisation by AGE. The template was 5-fold serially diluted. (**A**) CRISPR bulk assay. (**B**) AGE visualisation. M, DNA Ladder H1 (100~1000 bp); NTC, no template control; Lanes 1–6, 9 × 10^4^, 1.8 × 10^4^, 3.6 × 10^3^, 7.2 × 10^2^, 144, and 29 copies/μL plasmid.

To achieve rapid detection, we aimed to reduce the number of cycles and thereby decrease the reaction time. The assay was tested with 35, 32, 29, and 26 cycles. A five-fold serial dilution of plasmid was used as a DNA template, and the amplified product was added to the CRISPR reaction system and incubated for 15 min (Figure S1). The fluorescence signal in the tube and the AGE all show that there was no significant change in sensitivity to T-nos detection at amplification of the plasmid DNA using 35, 32, and 29 cycles, while there was a decrease in sensitivity at 26 cycles (Figure 4A,B). Based on the experimental results, 29 cycles were considered optimal. For the purposes of portability and rapid detection, we felt that in-tube fluorescence signal detection was more in line with our objectives. In addition, the final 5 min extension of the PCR amplification did not affect CRISPR detection (Figure S2). In summary, the reaction conditions for the standard start 29 cycles of 98 °C for 10 s, 59 °C for 15 s, and 68 °C for 10 s. After the reaction, the amplification products were directly placed in the in-tube CRISPR detection system, and the whole system completed detection within 50 min.

### 3.4. Specificity and Sensitivity of Detection

**Figure 5:**
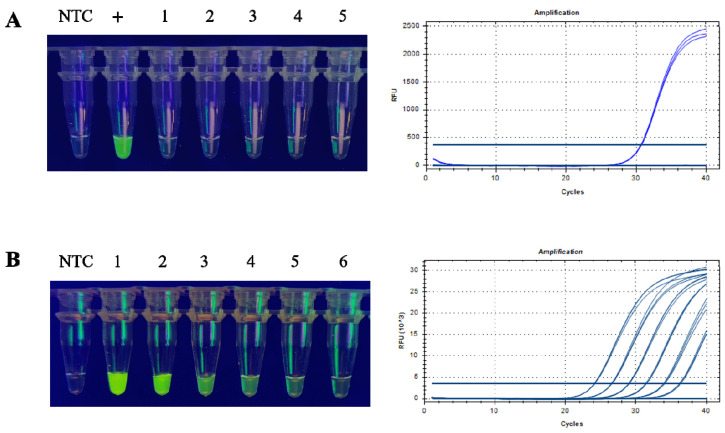
Specificity and sensitivity analyses. (**A**) Specificity evaluation of the CRISPR/Cas12a assay for T-nos detection. The same samples were measured by qPCR. +, positive samples; Lanes 1–5, non-GM cotton, non-GM soybean, non-GM rice, non-GM canola, non-GM corn. (**B**) Sensitivity evaluation of the CRISPR/Cas12a assay for T-nos detection. The same samples were measured by qPCR. Lanes 1–6, 1.88 × 10^4^, 3.75 × 10^3^, 7.5 × 10^2^, 1.5 × 10^2^, 30, and 6 copies/μL GM soybean. NTC, no template control.

To test the specificity of the established method, DNA from transgenic soybean ZUTS-33 and other non-transgenic plant samples (both concentrations were adjusted to ~800 copies/μL) were used for PCR amplification, and amplification products were added to the CRISPR system for T-nos detection. The results showed that only the amplification products of DNA from transgenic soybean samples resulted in visible fluorescence in the CRISPR system, while no significant fluorescence signal was generated for other samples or blank controls (Figure 4A, Figure 5A and Figure S3). The results indicate that the method established in this study for detecting NOS terminator sequences is specific. For comparison, the real-time fluorescence PCR system was used to amplify the same batch of DNA samples, and transgenic samples were found to have a low cycle threshold (Ct) value (30.69 ± 0.05), with positive amplification. Other non-transgenic DNA gave negative results.

In the sensitivity assays, PCR amplification was performed using a 5-fold serial dilution of transgenic soybean DNA as a template, and amplification products were incubated in the CRISPR reaction system. The results showed that fluorescence signals were observed at DNA concentrations of 1.88 × 10^4^, 3.75 × 10^3^, 7.5 × 10^2^, 1.5 × 10^2^, and 30 copies/μL for all samples (Figure 5B and Figure S4). The fluorescence signal tended to decrease with decreasing concentration, while the signal could not be observed at a DNA sample concentration of 6 copies/μL. This indicates that our conventional PCR-based CRISPR assay could detect samples down to 60 copies in the reaction. The same samples were detected by qPCR, and with the decrease in DNA concentration, its CT value gradually increases. Our CRISPR/Cas12a assay can detect as little as 60 copies of DNA sample within the reaction. It provides good sensitivity, and also has the advantages of convenience and fast detection.

### 3.5. Detection of Actual Samples

**Figure 6:**
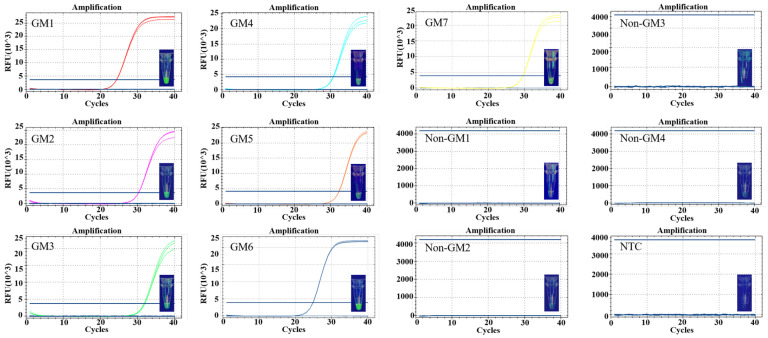
Testing of actual samples. Detection of T-nos components in 11 soybean DNA samples by CRISPR fluorescence detection system and qPCR.

Seven different batches of transgenic soybean samples ZUTS-33 (Transgenic *g10-epsps* gene) with NOS terminators provided by Zhejiang University and four non-transgenic soybean samples purchased from a market were tested using the conventional PCR/CRISPR bulk assay system, and the results were compared with those of the qPCR assay, to verify whether the established assay had the ability to detect T-nos components in crops (Figure 6). The established assay yielded fluorescence signals for T-nos in all samples, and the fluorescence intensity was generally consistent with the Ct value from qPCR (Table S2), indicating that the assay could detect NOS terminators effectively.

## 4. Discussion

In recent years, there has been worldwide concern regarding the safety of GM foods. GM plants are cultivated worldwide due to their ability to save time and labour, high quality, and high yield. Although a variety of GM crops have been developed in China, no GM crops have been commercialised. GM crops must be thoroughly assessed before they can be consumed by humans due to possible unintended effects [23,24,25,26]. This calls for more effective monitoring and stricter regulation of transgenic plants. In the present study, NOS terminators in transgenic plants were detected using CRISPR/Cas12a as a detection tool based on transgenic principles [27,28,29,30]. As a transcriptional regulatory sequence, the NOS terminator can serve as an important indicator to determine whether there are transgenic components. Recently, many convenient and rapid assays have emerged, such as RPA, LAMP, transcription-mediated amplification (TMA), and cross-priming amplification (CPA) [31,32,33,34,35,36]. However, due to expensive reagents and a complex primer design, they have not become established for widespread transgenic detection, and PCR instead remains the primary method of detection [37]. Conventional PCR is sensitive and inexpensive, and not easily contaminated, but the reaction time is long, and the results are not directly observable [38]. Therefore, improving the method to allow observable results outside the laboratory is a major aim.

The PCR-CRISPR/Cas12a system proposed in this study is based on the RNA-mediated DNA targeting of CRISPR effector protein Cas12a trans-cleavage as a modality, and it uses Cas12a-cleaved ssDNA (one end contains a fluorescent moiety and the other end contains a quenched group). By combining conventional PCR amplification with a CRISPR/Cas12a-based batch reaction system, PCR and CRISPR/Cas12a are coupled to provide dual-specific recognition of target genes, greatly improving detection specificity and sensitivity. The optimisation experiments for PCR-CRISPR/Cas12a showed that the higher the number of amplification reaction cycles, the higher the concentration of amplicons obtained. However, the CRISPR assay achieved more convenient amplicon detection than traditional AGE, indicating that combining the two can achieve better detection capability. The whole system can achieve a final detection limit for actual samples of ~60 copies in the reaction within 50 min, and at the same time guarantees specificity. It provides a fast and sensitive assay of NOS terminator.

Of course, our detection method also has some shortcomings and needs to be improved. For example, we have many detection steps, so we still rely on an operator with experimental operation ability. For this reason, we are also considering the development of simpler detection methods, such as the design of premix, the development of test strips, etc. The main purpose of our work is to establish an accurate, rapid, and cost-effective method for T-nos detection. However, the detection limit of 60 copies in the reaction still has deficiencies [39]. Therefore, in the follow-up work, we will also explore more efficient amplification and detection methods based on a low cost and short time to improve sensitivity. In addition, we will test more GM and non-GM crops to further evaluate the specificity of our method. A limitation of our detection is that not all GM events contain the target sequence used here. Beyond T-nos, we envision that the developed PCR-CRISPR/Cas12a assay will be extended to other transgenic elements, such as CaMV 35S promoter, E9 terminator, and T-35S terminator.

Although real-time PCR is still the main method for transgenic detection, it requires thermal cycling, an excitation light source, and a fluorescence detection device, which makes it difficult to achieve rapid detection in the field. The cost was calculated according to the consumables and instruments used in our laboratory. In terms of the price of consumable material and reagents, our method is basically the same as qPCR, but the real-time thermocyclers are often very expensive (more than 200,000 CNY), while with no more than half of this price, a lab can be equipped with all the instruments required to perform our method. Although the developed assay still requires instrumentation, there are several portable PCR instruments on the market, such as the Palm F1-12 handheld PCR instrument (Ahram Biosystems, Korea). It is only the size of a palm and uses a lithium battery to power it. The portable blue light excitation lamp can also be carried around, making it possible to get out of the laboratory and into the field. The method is not very demanding, it is cost-effective, rapid, and suitable for the mass detection of transgenic components in plants in the field.

## 5. Conclusions

In conclusion, our assay targets the exogenous NOS terminator sequence in transgenic plants, and it represents a simple, rapid, and cost-effective CRISPR/Cas12a-based field visualisation method for detecting T-nos. Compared with qPCR, the assay can save time while retaining high sensitivity and specificity. Thus, our method provides a new tool for transgene detection.

## Data Availability

Data are contained within the article.

## Supplementary Materials

The following supporting information can be downloaded at: https://www.mdpi.com/article/10.3390/foods12030615/s1, Figure S1: Endpoint fluorescence graphs for the T-nos detection with various incubation time (5, 10, 15 and 20 min) at 37 °C; Figure S2: Effect of PCR with or without the final 5 min extension on CRISPR reaction; Figure S3: Specificity evaluation of the CRISPR/Cas12a assay for T-nos detection; Figure S4: Two other repetitions based on sensitivity test; Table S1: The list of all used sequences in this study; Table S2: Ct values of qPCR for T-nos detection from 11 soybean DNA samples.
