# Supplementary material for: An Accurate, Rapid and Cost-Effective Method for T-nos Detection Based on CRISPR/Cas12a
Source: Foods. 2023 Feb 1;12(3):615. doi: 10.3390/foods12030615 (PMC9914525; doi:10.3390/foods12030615)
Supplement: Supplementary file 1 [file foods-12-00615-s001.zip › foods-2123937-supplementary.pdf]

Supplementary data

Table S1. The list of all used sequences in this study.

Primer name	Sequence (5′-3′)
ssDNA-FQ reporter	FAM-TTTTTTTTTTT-BHQ1
crRNA-NOS 1	UAAUUUCUACUCUUGUAGAUAAUACGCGAUAGAAAACAAA
crRNA-NOS 2	UAAUUUCUACUCUUGUAGAUCUGUUGAAUUACGUUAAGCA
crRNA-NOS 3	UAAUUUCUACUCUUGUAGAUGGCAAUAAAGUUUCUUAAGA
NOS-QF_SN	TTGGCAATAAAGTTTCTTAAGATTGAAT
NOS-QR_SN	ACATGCTTAACGTAATTCAACAGAAATT
NOS-QP_SN	6-FAM-CTGTTGCCGGTCTTGCGATGATTATCAT-BHQ-1
NOS-F1	GAATCCTGTTGCCGGTCTTG
NOS-R1	TTATCCTAGTTTGCGCGCTA
NOS-F2	ATCGTTCAAACATTTGGCA
NOS-R2	ATTGCGGGACTCTAATCATA

Table S2. Ct values of qPCR for T-nos detection from 11 soybean DNA samples.

**Samples**	**NTC**	**GM1**	**GM2**	**GM3**	**GM4**	**GM5**	**GM6**
CT value	-	24.37±0.02	30.28±0.01	31.88±0.11	30.69±0.1	32.28±0.04	24.83±0.04
**Sample**	**GM7**	Non-GM1	Non-GM2	Non-GM3	Non-GM4		
CT value	29.53± 0.05	-	-	-	-		

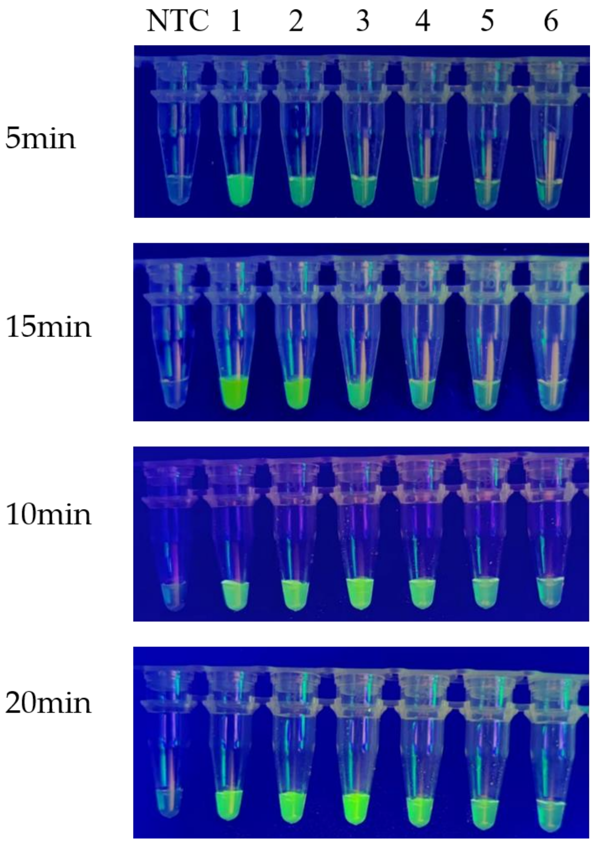

Figure S1. Endpoint fluorescence graphs for the T-nos detection with various incubation time (5, 10, 15 and 20 min) at 37 °C. No obvious change in visual fluorescence after incubation for 15 minutes or 20 minutes.

NTC, no template control; Lanes 1–6, 9×10^4, 1.8×10^4, 3.6×10^3, 7.2×10^2, 144, and 29 copies/μL plasmid.

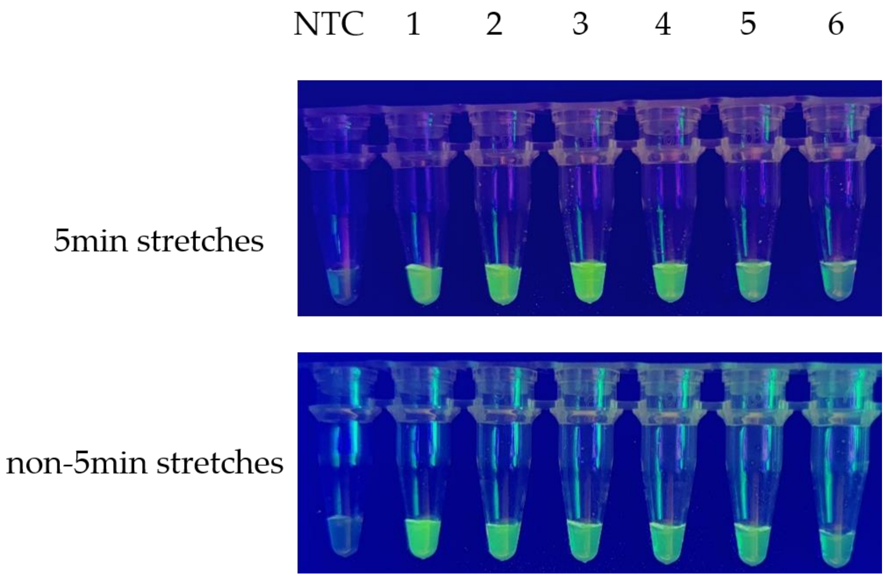

Figure S2. Effect of PCR with or without the final 5 min extension on CRISPR reaction. NTC, no template control; Lanes 1–6, 9×10^4, 1.8×10^4, 3.6×10^3, 7.2×10^2, 144, and 29 copies/μL plasmid.

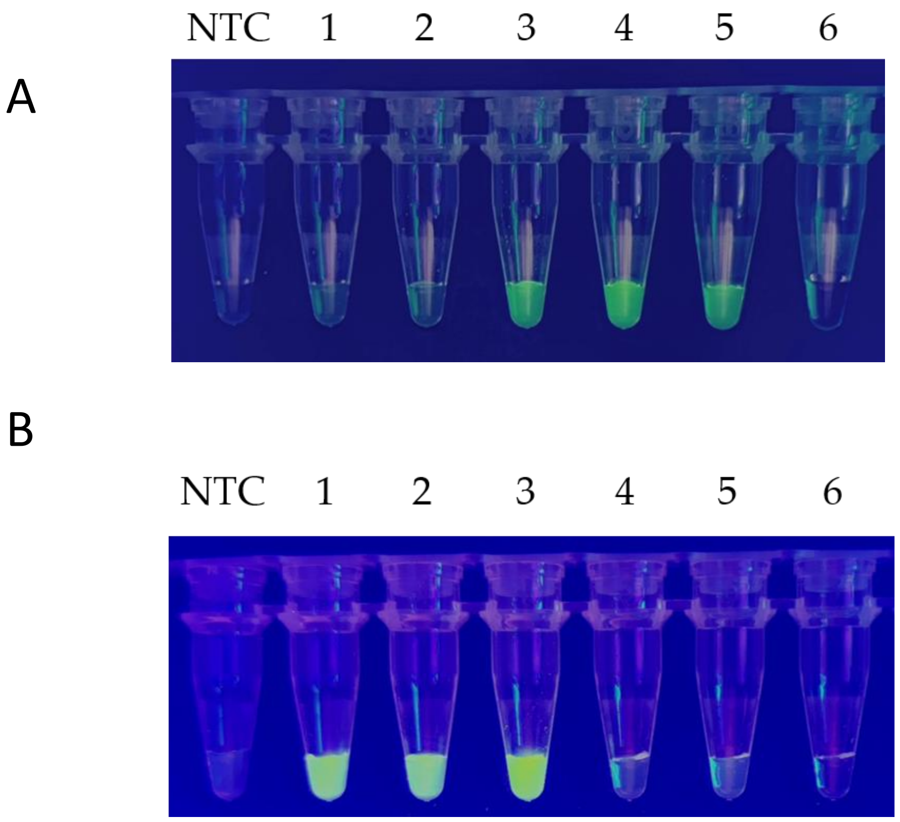

Figure S3. Specificity evaluation of the CRISPR/Cas12a assay for T-nos detection.
A:1-2: CaMV 35S promoter, T-35S terminator; 3-5: Three transgenic corn containing T-nos samples; 6: non-NOS GM soybean (MON89788).
B: 1-3: Three different DNA concentrations of GM soybean ZUTS-33; 4-6: Three different DNA concentrations of non-GM soybeans.

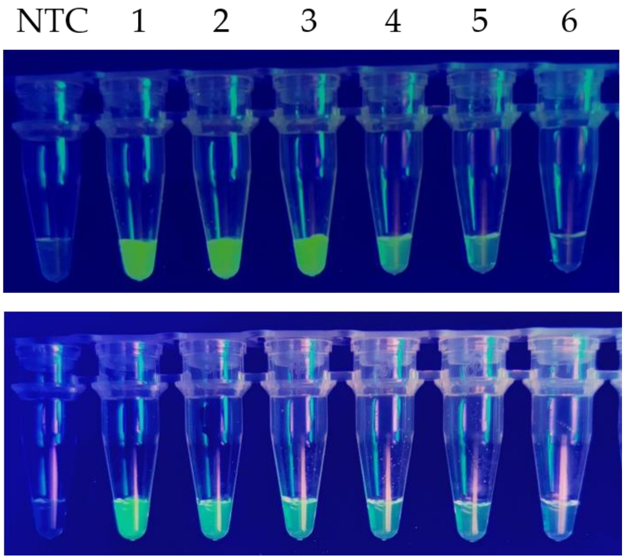

Figure S4. Two other repetitions based on sensitivity test.
